# Continuous apomorphine infusion modulates cortical hyperexcitability and abnormal plasticity in Parkinson's disease

**DOI:** 10.1177/1877718X261479077

**Published:** 2026-09-18

**Authors:** Maria Ilenia De Bartolo, Francesco Marchet, Sara Pietracupa, Carolina Cutrona, Flavia Aiello, Matteo Costanzo, Giorgio Leodori, Giorgio Vivacqua, Antonella Conte, Giovanni Fabbrini, Alfredo Berardelli, Nicola Modugno, Daniele Belvisi

**Affiliations:** 1 Department of Radiological Sciences, Oncology and Anatomical Pathology, 9311 Sapienza University of Rome, Rome, Italy; 2 IRCCS Neuromed, Pozzilli (IS), Italy; 3 Department of Human Neurosciences, Sapienza University of Rome, Rome, Italy; 4 Department of Neurology and Stroke Unit, 9262 San Giovanni Addolorata Hospital, Rome, Italy; 5 Department of Neuroscience, 9289 Istituto Superiore di Sanità, Rome, Italy; 6 Department of Experimental Morphology and Microscopy Integrated Research Center (PRAAB), 220431 Campus Biomedico University of Rome, Rome, Italy

**Keywords:** Parkinson's disease, apomorphine, transcranial magnetic stimulation, cortical excitability, cortical plasticity, kinematic analysis

## Abstract

**Background:**

Several studies have demonstrated the clinical benefit of apomorphine subcutaneous infusion in Parkinson's disease (PD). However, it remains unclear whether apomorphine infusion also modulates the abnormal motor cortical excitability and plasticity that contribute to PD pathophysiology and progression.

**Methods:**

We investigated the effects of continuous apomorphine infusion on cortical excitability and plasticity in patients with moderate-advanced PD, assessed at baseline and after 1, 12, and 24 weeks of treatment. Transcranial magnetic stimulation (TMS) was used to evaluate: i) motor cortex excitability (via input-output (I-O) recruitment curves and motor evoked potentials (MEPs) at 100–140% of resting motor threshold); ii) intracortical inhibition/facilitation (SICI; ICF), and plasticity using intermittent theta-burst stimulation (iTBS). Kinematic analysis of finger movements quantified bradykinesia. Eighteen healthy controls (HC) underwent baseline assessments for comparison.

**Results:**

At baseline, PD patients showed increased cortical excitability and reduced iTBS-induced plasticity compared to HC. After 24 weeks of continuous apomorphine infusion, cortical excitability decreased (reduced I-O slope and MEP amplitudes at 120–140% rMT), and plasticity improved (greater MEP facilitation 30 min post-iTBS). Clinically, motor and non-motor symptoms improved paralleled by a significant reduction in oral levodopa dose. Changes in excitability correlated with changes in motor performance.

**Conclusions:**

We found that continuous apomorphine infusion was associated with progressive modulation of selected cortical markers in PD, in parallel with clinical improvement. These findings also highlight the potential value of cortical markers for monitoring treatment response in advanced PD.

## Continuous apomorphine infusion may rebalance brain activity and improve movement in advanced Parkinson’s disease

In people with more advanced Parkinson’s disease, the movement areas of the brain can become “overactive” and less able to adapt, which may worsen symptoms and contribute to motor fluctuations (periods when medication works well and periods when it wears off). Apomorphine is a dopamine-like drug that can be delivered continuously through a small pump under the skin. Continuous delivery may provide steadier symptom control than taking pills at intervals. In this study, we asked whether continuous apomorphine infusion also changes the activity of the brain’s movement control area. We followed people with moderate-to-advanced Parkinson’s disease before starting the infusion and then after 1 week, 12 weeks, and 24 weeks of treatment. We measured brain activity using non-invasive magnetic stimulation of the scalp and recorded muscle responses. We also used motion tracking to measure finger movement speed and timing, and we assessed motor and non-motor symptoms with standard clinical scales. A group of healthy volunteers was tested once for comparison. Before treatment, patients showed higher-than-normal brain excitability and reduced ability of the brain to “learn” or adjust after stimulation. After 24 weeks of continuous apomorphine infusion, brain excitability decreased and the brain’s ability to adapt improved. These changes happened alongside better motor performance, improvement in several clinical measures, and a reduction in the daily dose of oral levodopa. Overall, the findings suggest that continuous apomorphine infusion may improve symptoms partly by normalizing how the movement cortex works.

## Introduction

The onset and progression of Parkinson's disease (PD) are driven by complex pathophysiological mechanisms involving widespread alterations in basal ganglia–cortical circuits. Transcranial magnetic stimulation (TMS) offers a non-invasive approach to investigate the functional state of the primary motor cortex (M1), providing insights into cortical excitability, synaptic plasticity, and the balance between inhibitory and facilitatory mechanisms. In patients with PD, TMS studies have demonstrated an abnormal M1 activity, characterized by increased cortical excitability and reduced cortical inhibition and plasticity.^1–4^ Several studies have also demonstrated that these alterations are relevant for the pathophysiology of both motor symptoms and motor complications.^5–10^

Previous evidence showed that dopaminergic therapy with oral levodopa can transiently restore cortical inhibition.^11–14^ Acute levodopa can also restore, at least in part, cortical excitability and plasticity in early disease stages, but not in patients with advanced PD and motor fluctuations.^7–10,15–19^ Conversely, continuous dopaminergic stimulation with intrajejunal levodopa-carbidopa gel has been shown to re-engage mechanisms of cortical plasticity even in patients with advanced disease.^20^ These effects, however, remain critically dependent on presynaptic dopaminergic integrity, and the progressive loss of dopaminergic terminals may limit the long-term efficacy of levodopa-based therapies.

Apomorphine, a potent dopamine agonist with affinity for both D1- and D2-like receptors, bypasses presynaptic mechanisms and directly stimulates postsynaptic dopamine receptors.^21–23^ When administered via continuous subcutaneous infusion, apomorphine provides sustained dopaminergic stimulation, potentially mimicking physiological dopamine tone more effectively than pulsatile therapies.^24–29^

Despite its established clinical efficacy in reducing motor fluctuations and off-periods, the neurophysiological effects of continuous apomorphine infusion on cortical excitability and plasticity remain unknown, as they have not been previously investigated.

To address this knowledge gap, we investigated the effects of subcutaneous continuous apomorphine infusion on cortical excitability, inhibition, facilitation, and plasticity in patients with moderate to advanced PD, using a multimodal TMS protocol. This included assessments of input–output recruitment curves, short-interval intracortical inhibition (SICI), intracortical facilitation (ICF), and responses to intermittent theta burst stimulation (iTBS). Furthermore, to objectively quantify motor improvement, we employed kinematic analysis of finger movement performance, a sensitive marker of bradykinesia. Neurophysiological and kinematic assessments were conducted at baseline (prior to apomorphine initiation) and at 1, 12, and 24 weeks following the start of continuous infusion.

By integrating neurophysiological and behavioral metrics, this study aims to determine whether the clinical improvements associated with continuous apomorphine infusion are paralleled by restorative effects on motor cortex function.

## Materials and methods

### Participants

Twenty-one patients with PD and 18 age- and sex-matched healthy controls (HC) were consecutively enrolled from the Movement Disorders Centers at IRCCS Neuromed (Pozzilli, IS) and the Department of Human Neurosciences, Sapienza University of Rome (Rome, RM). Demographic and clinical data are shown in Table 1. Inclusion criteria for PD were: a diagnosis of PD^30^ and a moderate-advanced disease stage with a clinical indication for continuous subcutaneous apomorphine infusion both confirmed by a movement disorder expert. Exclusion criteria for PD included the presence of other neurological or psychiatric disorders, as well as any contraindications to undergoing TMS protocols. HC were enrolled among non-consanguineous patients’ relatives only for baseline evaluation. Exclusion criteria for HC were: the presence of any neurological or psychiatric disorders; the assumption of drugs known to induce parkinsonism; contraindications to undergoing TMS protocols.

**Table 1.** Clinical and demographic data.

	Age	Sex	Weight (Kg)	Height (cm)	Disease duration (years)	Hoehn & Yahr
PD	62.3 ± 2.1	8 females	64.9 ± 4.2	162.3 ± 2.9	13 ± 1.5	2.9 ± 1.5
HC	61.7 ± 1.8	11 females	/	/	/	/

Abbreviations: PD: Parkinson's disease; HC: healthy controls.

### Clinical assessment

Comprehensive clinical evaluations were performed at baseline and included both motor and non-motor domains. Disease stage was assessed using the Hoehn and Yahr (H&Y) scale, while disease severity was evaluated with the Movement Disorder Society-Unified Parkinson's Disease Rating Scale (MDS-UPDRS), including Parts I (non-motor experiences of daily living), II (motor experiences of daily living), III (motor examination), and IV (motor complications). Non-motor symptoms were assessed with the Non-Motor Symptoms Scale (NMSS), and global cognitive function was evaluated using the Montreal Cognitive Assessment (MoCA). Patient-reported quality of life was measured with the 39-item Parkinson's Disease Questionnaire (PDQ-39), while the Non-Motor Fluctuation Assessment questionnaire (NoMoFA) was used to capture patient-perceived fluctuations in non-motor symptoms. The levodopa equivalent daily dose (LEDD) was calculated to reflect oral dopaminergic therapy only and did not include the contribution of continuous subcutaneous apomorphine infusion. This approach was adopted to quantify changes in oral dopaminergic medication following the initiation of apomorphine therapy. Therefore, LEDD values represents an index of oral dopaminergic treatment rather than total dopaminergic load.

### TMS assessment

Transcranial magnetic stimulation (TMS) was performed using a Magstim 200 stimulator (Magstim, Whitland, UK) equipped with a 70-mm figure-of-eight coil. The TMS coil was positioned tangentially to the scalp over the primary motor cortex (M1), with the handle oriented posteriorly and laterally at approximately 45° from the midline to induce a posterior–anterior current in the cortex. Stimulation was delivered over M1 corresponding to the representation of the first dorsal interosseous (FDI) muscle (“motor hotspot”) on the more affected (bradykinetic) side in PD and on the left side in HC. Coil position and orientation were carefully monitored throughout the experiment to ensure consistency across trials and timepoints. Resting motor threshold (rMT) was determined for each participant, and *corticospinal excitability* was assessed by recording motor evoked potentials (MEP) at stimulus intensities corresponding to 100%, 120%, and 140% of the rMT to generate individual input-output recruitment curves.^31^

Intracortical inhibitory and facilitatory circuits were evaluated using paired-pulse paradigms. *Short-interval intracortical inhibition (SICI)* and *intracortical facilitation (ICF)* were tested using a subthreshold conditioning pulse at 80% rMT followed by a suprathreshold test stimulus at 120% rMT.^32–34^ Interstimulus intervals were set at 3 ms for SICI and 10 ms for ICF.

For each stimulation condition, a fixed number of trials was collected to ensure reliability of the measurements. Specifically, for input–output curves, MEP amplitudes were obtained by averaging responses across 10 stimuli at each intensity level. For paired-pulse paradigms (SICI and ICF), conditioned responses were averaged across 10 trials for each interstimulus interval.

*Cortical plasticity* was assessed using intermittent theta-burst stimulation (iTBS), applied via a Magstim SuperRapid stimulator (Magstim, Whitland, UK) with the same coil type.^35^ To evaluate the effects of iTBS on motor cortical excitability, 20 MEPs were recorded at 120% of the active motor threshold (aMT) before stimulation, and at 5, 15 and 30 min post-stimulation.^6^ Electromyographic (EMG) recordings were obtained from the FDI muscle using disposable Ag/AgCl surface electrodes in a belly-tendon montage. EMG signals were filtered (bandpass 10–1000 Hz), amplified (×1000) using a Digitimer D360 amplifier (Digitimer Ltd, UK), and digitized at 5 kHz with a CED 1401 interface (Cambridge Electronic Design, UK) for offline analysis. Peak-to-peak MEP amplitudes were averaged across trials. Trials were excluded if baseline EMG activity exceeded 100 µV within the 500 ms preceding the TMS pulse.

Due to the longitudinal design and the nature of the intervention, assessors were not blinded to the treatment timepoint. However, all neurophysiological assessments were performed using standardized acquisition protocols to minimize potential bias.

### Kinematic assessment

Kinematic evaluation of finger movement was performed using the SMART motion capture system (BTS Engineering, Milan, Italy), equipped with three high-resolution infrared cameras. Reflective markers were positioned on anatomical landmarks, including the distal phalanx and the metacarpophalangeal (MCP) joint of the index finger, as well as on the first and fifth metacarpal bones, to accurately track finger motion. The SMART Analyzer software was used to quantify the displacement of the index finger around the MCP joint. Movement parameters were extracted and expressed as: (1) range of motion (ROM), defined as the mean angular excursion in degrees; (2) movement time (TIME), calculated as the average time in seconds required to complete a single abduction cycle; and (3) angular velocity (VELOCITY), reported as the mean speed in degrees per second.^6,36,37^ All recordings were conducted on the most bradykinetic hand for each patient (as identified by clinical examination) and on the left hand in HC.

### Study design

Clinical, neurophysiological, and kinematic assessments were conducted at four time points in PD patients: one week before initiation of continuous subcutaneous apomorphine infusion (T0), and at 1 week (T1), 12 weeks (T2), and 24 weeks (T3) after treatment initiation. At baseline (T0), patients were assessed while on treatment, in the context of their optimized oral dopaminergic regimen. Because all patients were receiving levodopa, neurophysiological assessments were performed 1 h after levodopa administration at all time points. From T1 to T3, all assessments were performed while patients were receiving continuous subcutaneous apomorphine infusion in combination with oral therapy, 5 h after activation of the apomorphine pump and 1 h after oral levodopa administration.

### Statistical analysis

Statistical analyses were conducted using GraphPad Prism (version 9) and IBM SPSS Statistics (version 30). Continuous variables were inspected for normality using the Shapiro–Wilk test and visual inspection of histograms and Q–Q plots. According to the results of the normality test, the Mann-Whitney U test was used to verify differences in neurophysiological and kinematic features between PD patients and HC at baseline. The Friedman test was used to evaluate in PD patients longitudinal changes in clinical, neurophysiological, and kinematic measures across time points. When significant effects were detected, post hoc pairwise comparisons were performed. For correlation analyses, changes in clinical scales, kinematic features, and neurophysiological measures were quantified using delta scores, calculated as the difference between values at T3 and T0. All variables were standardized (z-scores) prior to analysis. Correlation analyses were restricted to those behavioral (clinical and kinematic) and TMS measures that showed significant longitudinal changes following apomorphine treatment, as identified by Friedman tests. Associations of interest were then assessed using partial correlations, controlling for age and disease duration to account for potential confounding effects. To control for multiple comparisons and reduce the risk of false-positive findings, p-values were adjusted using the Benjamini–Hochberg false discovery rate (FDR) procedure. Given the small sample size, the robustness of the observed associations was further evaluated using leave-one-out sensitivity analyses, iteratively repeating each correlation after excluding one subject at a time.

## Results

### Participants

Fifteen out 21 PD patients completed the follow-up at T3 and were therefore considered for statistical analysis. Among the six patients who did not complete the study, one discontinued because of cognitive worsening, one due to severe orthostatic hypotension, two because of persistent nausea, and two voluntarily withdrew from the study before completing the follow-up.

Baseline differences between PD and HC for TMS and kinematic studies

MEP amplitude at **100% rMT** was significantly higher in PD (median: 0.14, IQR:0.09) vs HC (median: 0.07, IQR: 0.1) (U = 214.00, p = .013), as well as at **120% rMT** (PD median: 0.76, IQR: 0.65; HC median: 0.46, IQR: 0.37; U = 178.00, p = .019) and at **140%** **rMT** (PD median: 1.8, IQR: 1.5; HC median: 0.92, IQR: 0.7; U = 165.00, p = .001). The increased excitability in PD vs HC was further confirmed by the **slope** of input-output curve which was significantly higher in PD (median: 0.036, IQR: 0.003) compared to HC (median: 0.023, IQR: 0.01) (U = 170.00, p = .012). No significant differences were observed in SICI and ICF between PD and HC.

Motor cortical plasticity was significantly reduced in PD compared to HC, as shown by the lowered increase of normalized MEP amplitude (%) **5 min post iTBS** paradigm in PD (median: 90.63%, IQR: 70.26) vs HC (median: 114.72%, IQR: 13.64) (U = 69.50, p = .021), as well as **15 min post iTBS** (PD median: 112.59%, IQR: 88.39; HC median: 140.35%, IQR: 33.82; U = 78.00, p = .04) and **30 min post iTBS** (PD median: 89.73%, IQR: 72.70; HC median: 128.57%, IQR: 38.24; U = 73.00, p = .029). The variables **VELOCITY** (PD median: 88 degrees/sec, IQR: 59; HC median: 292 degrees/sec, IQR: 230; U = 13.00, p < .001) and **ROM** (PD median: 17.59 degrees, IQR: 6.63; HC median: 28.36 degrees, IQR 7.49; U = 196.0, p < .001) of kinematic performance were significantly lower in PD compared to HC. No significant differences were detected in TIME between PD and HC.

Longitudinal changes of clinical features from T0 to T3.

**Figure 1.**
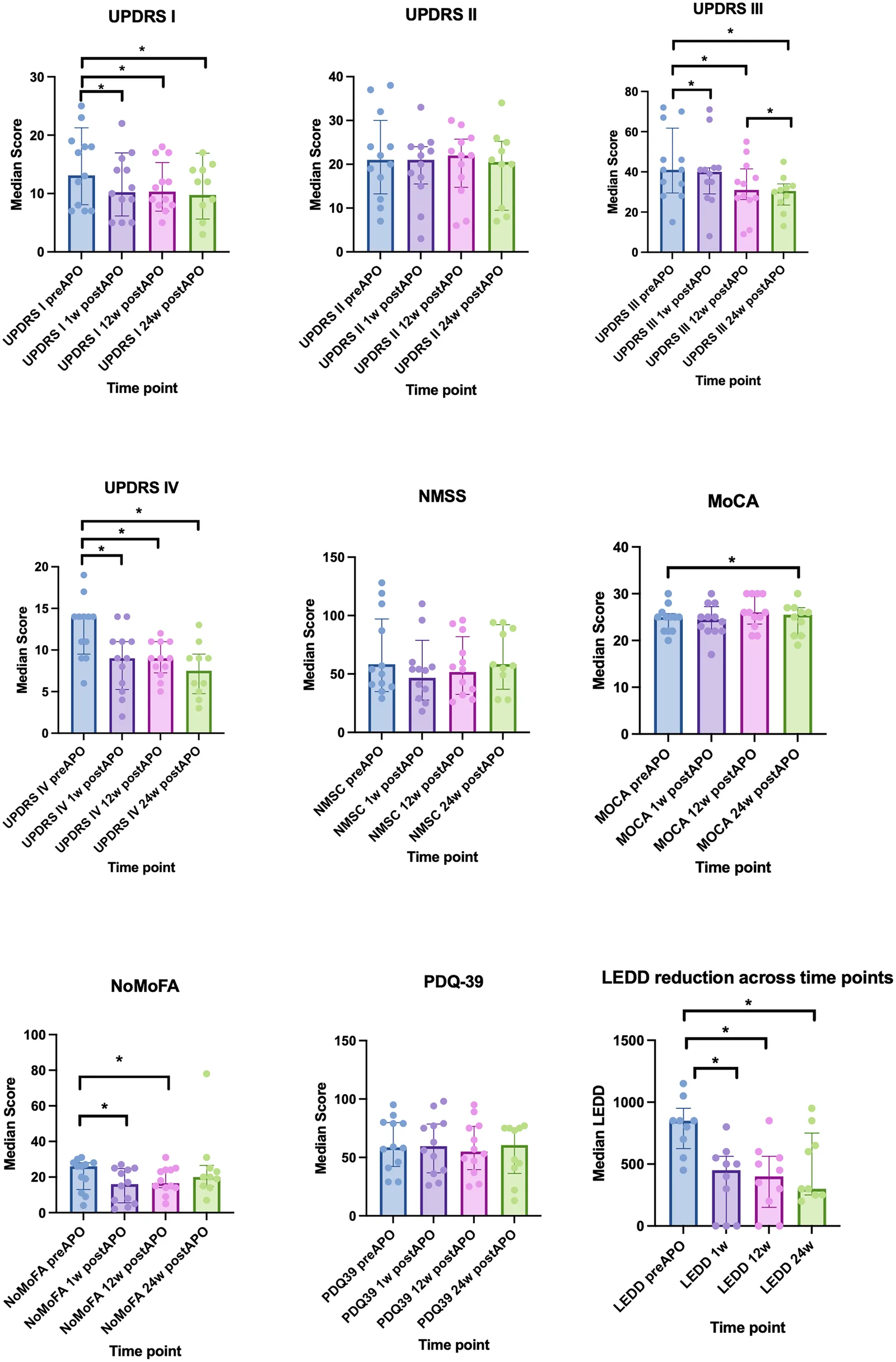
Longitudinal changes in clinical scales and dopaminergic treatment in patients with Parkinson's disease during continuous apomorphine infusion. Bar graphs show group median and individual data points for clinical scores assessed at baseline (preAPO), 1 week (1w), 12 weeks (12w), and 24 weeks (24w) after treatment initiation. UPDRS: Unified Parkinson's Disease Rating Scale; NMSS: Non-Motor Symptoms Scale; MoCA: Montreal Cognitive Assessment); NoMoFA: Non-Motor Fluctuation Assessment; PDQ-39: Parkinson's Disease Questionnaire, total score; LEDD: levodopa equivalent daily dose. Bars represent median ± interquartile range. Statistical significance is indicated as *.

A significant improvement was observed in **UPDRS I** (Chi-Square = 10.935, Kendall's W = 0.243, p = 0.012), **UPDRS III** (Chi-Square = 12.649, Kendall's W = 0.281, p = .005), **UPDRS IV** (Chi-Square = 9.065, Kendall's W = 0.201, p = .028), **MoCA** (Chi-Square = 11.321, Kendall's W = 0.252, p = .010), **NoMoFA** (Chi-Square = 9.969, Kendall's W = 0.222, p = .019) scores. In detail, UPDRS I significantly changed from T0 to T1 (Z = −3.068, r = 0.79, p = .002), T0 to T2 (Z = −2.658, r = 0.69, p = .008), T0 to T3 (Z = −2.493, r = 0.64, p = .013); UPDRS III from T0 to T1 (Z = −2.121, r = 0.55, p = .034), T0 to T2 (Z = −2.272, r = 0.59, p = .023), T0 to T3 (Z = −2.934, r = 0.76, p = .003), T2 to T3 (Z = −2.494, r = 0.64, p = .013); UPDRS IV from T0 to T1 (Z = −2.946, r = 0.76, p = .003), T0 to T2 (Z = −2.414, r = 0.62, p = .016), T0 to T3 (Z = −2.504, r = 0.65, p = .012); MoCA from T0 to T3 (Z = −2.204, r = 0.57, p = .027); and NoMoFA from T0 to T1 (Z = −3.062, r = 0.79, p = .002), from T0 to T2 (Z = −2.385, r = 0.62, p = .017). See Figure 1.

**LEDD** significantly decreased over time (Chi-Square = 8.838, Kendall's W = 0.2, p = .032), with post hoc analyses showing reductions from T0 to T1 (Z = −2.524, r = 0.65, p = .012), from T0 to T2 (Z = −2.207, r = 0.57, p = .027) and from T0 to T3 (Z = −2.214, r = 0.57, p = .027) (Figure 1). **Apomorphine** infusion rate did not significantly differ across time points. The median infusion rates were 1.7 mg/h (IQR: 1–2) at T1, 2.0 mg/h (IQR: 1–3) at T2, and 1.9 mg/h (IQR: 1–3.2) at T3.

Longitudinal changes of TMS features from T0 to T3.

**Figure 2.**
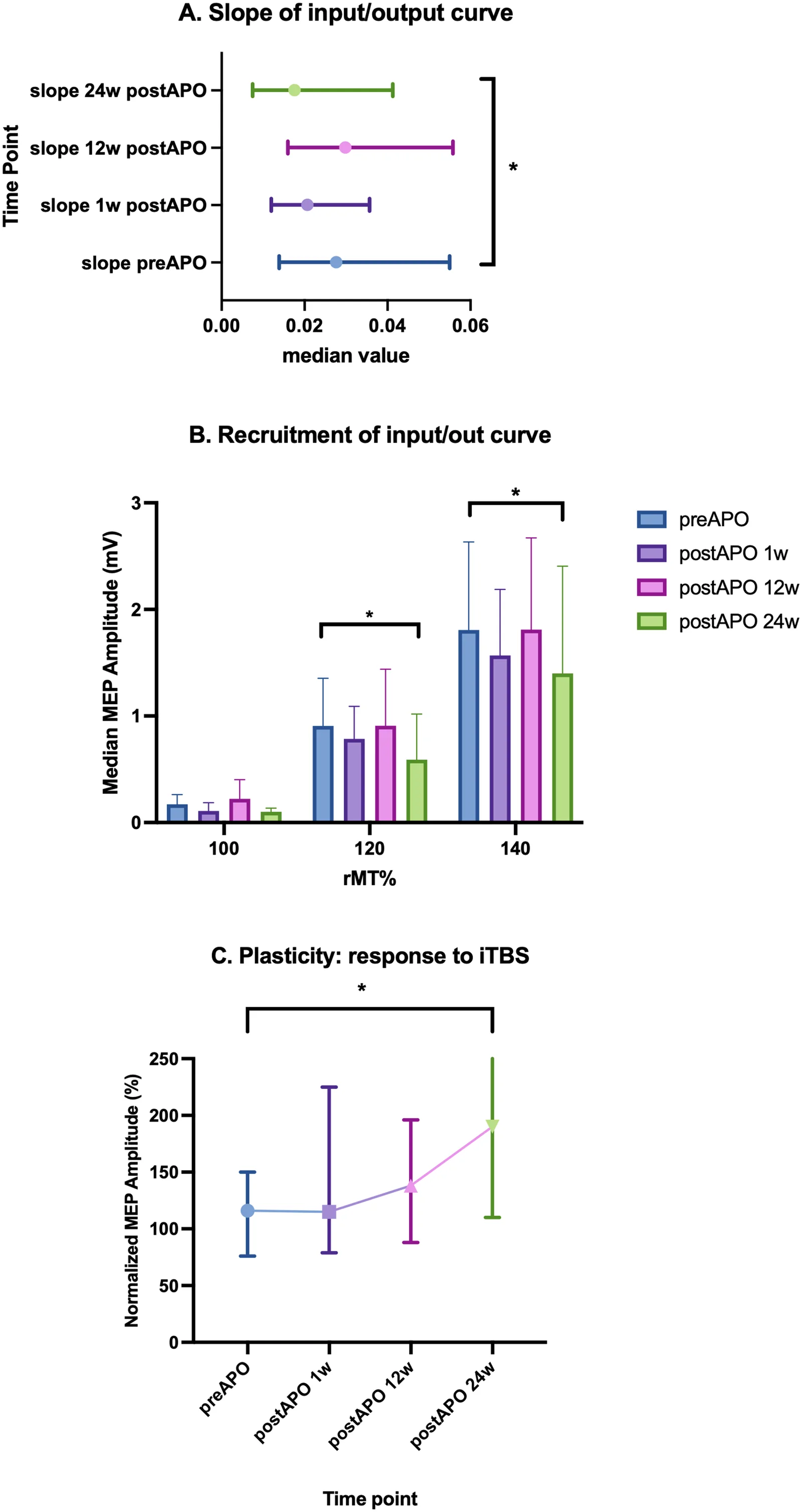
Longitudinal changes in neurophysiological features of motor cortex function. (a) Slope of the input–output (I–O) curve, reflecting corticospinal excitability, decreased significantly over time. (b) Mean MEP amplitude at 120% and 140% of the resting motor threshold (rMT) also decreased across time points, indicating normalization of cortical excitability. (c) Plasticity response to intermittent theta-burst stimulation (iTBS), expressed as MEP amplitude normalized to pre-iTBS baseline, increased significantly 30 min post-stimulation at 24 weeks (24w), indicating improvement of LTP-like plasticity. Bars and line plots show group median ± interquartile range. Statistical significance is represented as *.

The **slope** of the input–output curve significantly decreased over time (Chi-Square = 10.714, Kendall's W = 0.3, p = .013), with a notable reduction observed after apomorphine infusion from T0 to T3 (Z = −2.7, r = 0.7, p = .007) (Figure 2(a)). A significant decrease was also observed in the mean MEP amplitude at **120% rMT** stimulation intensity (Chi-Square = 14.7, Kendall's W = 0.3, p = .002), particularly from T0 to T3 (Z = −2.097, r = 0.5, p = .036). Similarly, mean MEP amplitude at **140% rMT** stimulation intensity showed a significant reduction (Chi-Square = 8.2, Kendall's W = 0.2, p = .042) from T0 to T3 (Z = −2.118, r = 0.5, p = .034) (Figure 2(b)). SICI and ICF did not significantly change across time points (Supplementary Materials, Figure S1).

Responses to the iTBS paradigm revealed a significant increase of MEP amplitude - normalized to pre-iTBS baseline - **30 min post iTBS** across different time points (Chi-Square = 8.769, Kendall's W = 0.2, p = .033). Indeed, post hoc analysis showed that normalized MEP amplitude at T3 was significantly higher than at T0 (Z = −2.934, r = 0.758, p = .003) (Figure 2(c)).

Longitudinal changes of kinematic performance from T0 to T3.

**Figure 3.**
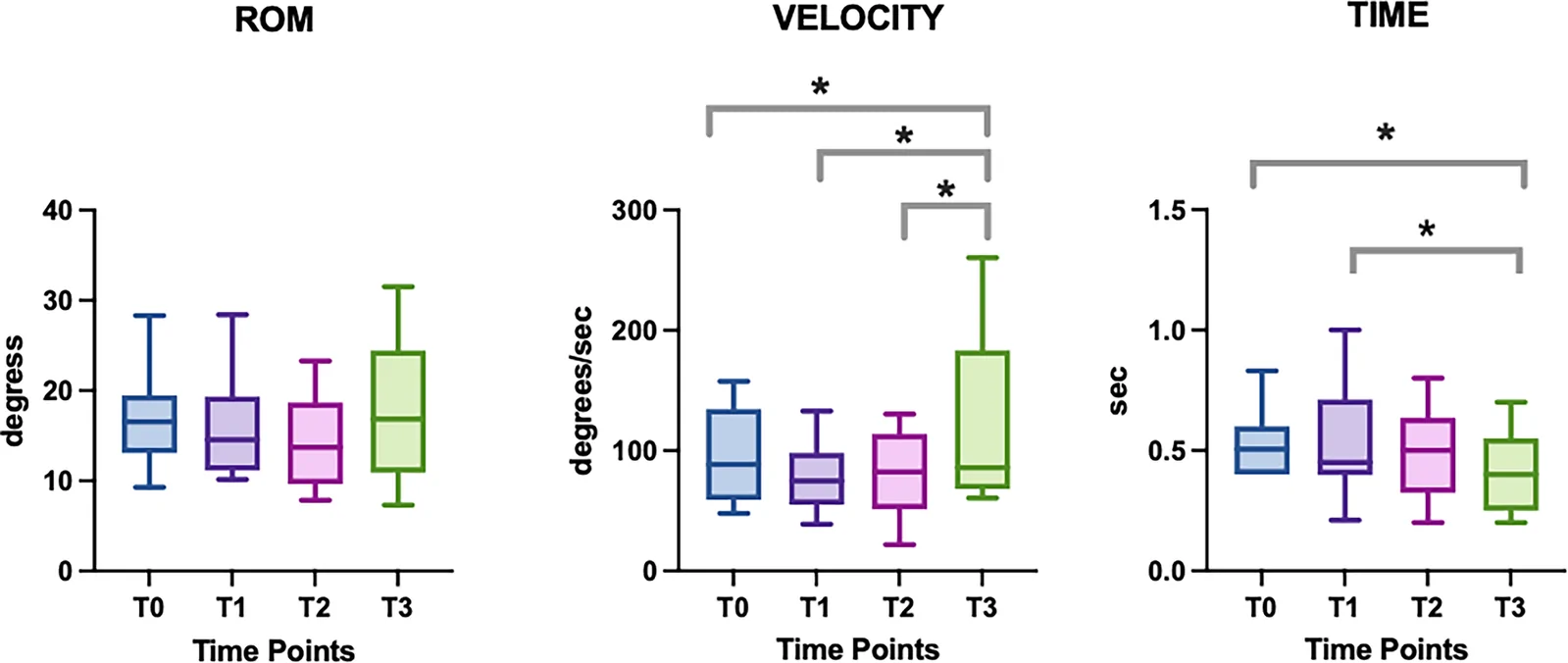
Longitudinal changes in kinematic performance of finger movement in patients with Parkinson's disease. Box plots represent the distribution of kinematic parameters at baseline (T0), and at 1 week (T1), 12 weeks (T2), and 24 weeks (T3) after initiation of continuous apomorphine infusion. ROM: Range of motion; VELOCITY: average speed of finger movement; TIME: time to complete one abduction cycle. Boxes indicate median and interquartile range; whiskers represent minimum and maximum values. Statistical significance is indicated as *.

A significant reduction in **TIME** required to perform finger movements was observed across time points (Chi-Square = 13.245, Kendall's W = 0.3, p = .004). Indeed, kinematic analysis revealed a significant decrease in movement time from T0 to T3 (Z = −2.043, r = 0.53, p = .041) and from T1 to T3 (Z = −2.246, r = 0.58, p = .025) (Figure 3). A significant increase in **VELOCITY** was also observed (Chi-Square = 8.500, Kendall's W = 0.2, p = .037), specifically from T0 vs T3 (Z = −2.432, r = 0.63, p = .015), from T1 vs T3 (Z = −2.794, r = 0.72, p = .005) and from T2 vs T3 (Z = −1.992, r = 0.51, p = .04) (Figure 3).

Clinical and neurophysiological correlations

**Figure 4.**
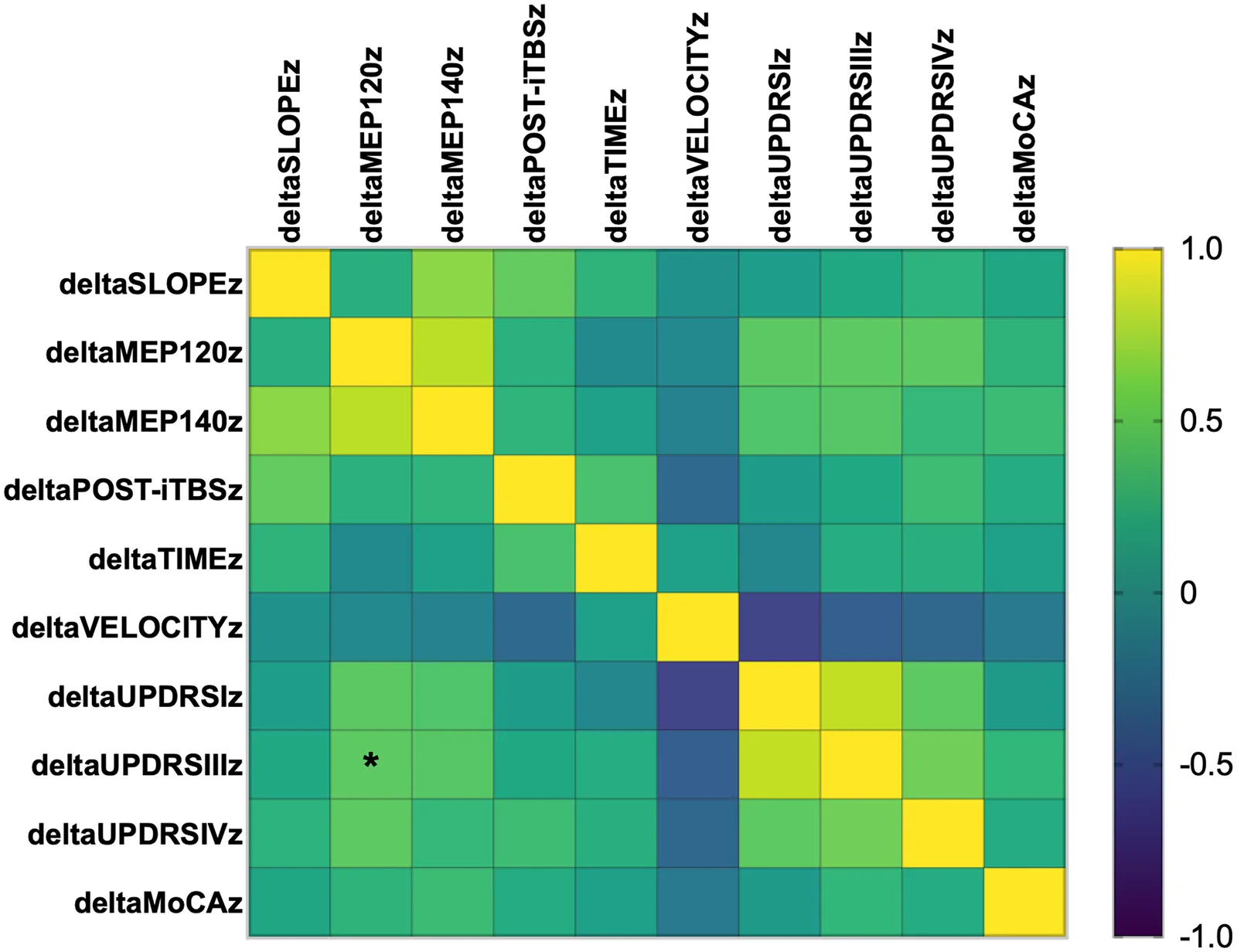
Heatmap showing correlations between changes in neurophysiological (TMS), clinical and kinematic measures. Correlations were computed using using z-scored delta values (T3–T0) and adjusted for age and disease duration. Warmer colors indicate stronger positive correlations, whereas cooler colors indicate weaker or negative correlations. Statistical significance was assessed using the Benjamini–Hochberg false discovery rate (FDR) correction for multiple comparisons. The asterisk (*) denotes correlations that remained significant after FDR correction.

After FDR correction, only the correlation between the change in MEP amplitude at 120% rMT and the change in UPDRS-III remained significant (r = 0.891, uncorrected p = 0.001, FDR-adjusted q = 0.020), thus meaning that a reduction in M1 cortical excitability (lower MEP amplitude at **120% rMT** from T0 to T3) was significantly associated with greater clinical improvement (lower **UPDRS-III** from T0 to T3). Other correlations showing nominal significance - namely those between delta MEP 120% rMT and delta UPDRS I (r = 0.704, p = 0.034), delta MEP 140% rMT and delta UPDRS III (r = 0.729, p = 0.026), delta slope of input-output curve and delta TIME kinematic feature (r = 0.707, p = 0.033), and delta post-iTBS normalized MEP amplitude and delta TIME kinematic feature (r = 0.787, p = 0.012) - did not survive correction for multiple comparisons (all q > 0.10) (Figure 4).

## Discussion

In the present study we found that PD patients, at baseline, presented M1 hyperexcitability and reduced plasticity compared to HC, consistent with prior TMS studies on PD patients.^1,2,5,6,8,9,38–42^ Specifically, PD patients had steeper MEP recruitment curves and blunted responses to plasticity induction (reduced MEP facilitation after iTBS) compared to HC, while no differences were detected in inhibitory and facilitatory mechanisms (SICI and ICF). Over 24 weeks of continuous apomorphine infusion, selected cortical abnormalities were significantly modulated. By week 24 from apomorphine initiation, PD patients exhibited a reduced input-output slope and smaller MEP amplitudes at suprathreshold intensities (120–140% rMT), indicating an improvement of cortical excitability. Concurrently, motor cortex plasticity improved, as shown by a greater MEP potentiation 30 min after iTBS at 24 weeks compared to baseline. However, no significant changes were observed in intracortical inhibitory and facilitatory measures (SICI and ICF). Clinically, patients experienced sustained motor improvement (lower UPDRS-III and IV scores and faster finger movement kinematics) alongside better non-motor outcomes (improved UPDRS-I, NoMoFA and MoCA scores) and a reduction in total dopaminergic medication dose. Overall, this study suggests a role of continuous dopaminergic stimulation via subcutaneous apomorphine infusion in ameliorating abnormal motor cortical excitability and plasticity in PD patients, in parallel with clinical improvements.

Continuous apomorphine infusion is an established therapy for advanced PD, and our results provide neurophysiological evidence underpinning its clinical benefits. PD patients in our study had markedly reduced motor severity, “OFF” time and motor fluctuations, reflected in lower UPDRS-III and IV scores and faster movement kinematics after apomorphine initiation. Importantly, continuous apomorphine infusion allowed for a substantial reduction in oral levodopa daily dose, while maintaining improved motor control. This aligns with previous open-label studies showing apomorphine infusion can sustain motor benefits and significantly lower the oral medication burden.^24–27,29,43–45^

Moreover, the improvement in UPDRS I scores across all follow-up time points, suggests a potential effect of apomorphine on non-motor domains. However, no significant changes were observed in the NMSS, and the reduction in NoMoFA scores was limited to the early phase (T1-T2), indicating that the impact of continuous apomorphine infusion on non-motor symptoms may be modest and variable. Although MoCA scores showed a significant improvement from baseline to T3, the overall effect on non-motor features appears less pronounced than that observed for motor symptoms, consistent with previous findings.^46–48^

The observed neurophysiological changes after apomorphine initiation (reduced cortical excitability and enhanced plasticity after 24 weeks) align with the concept that pulsatile vs. continuous dopaminergic stimulation have different effects on cortical circuits. Indeed, intermittent oral levodopa can transiently rescue impaired plasticity in early PD,^9^ but loses efficacy in advanced disease as pulsatile stimulation induces maladaptive changes in basal ganglia–cortical pathways.^7,10,15,49^ Moreover, PD patients with motor fluctuations display persistent cortical hyperexcitability that are only partially responsive to acute levodopa.^10^ On the other hand, a prior work with continuous intrajejunal levodopa (LCIG) showed that switching from oral dosing to continuous infusion in advanced PD patients improved abnormal motor cortex excitability, restored intracortical inhibition, and partially normalized LTP-like plasticity.^20^ Our results provide the first evidence that continuous apomorphine infusion, through direct activation of both D1 and D2 dopamine receptors, may ameliorate cortical excitability and plasticity, similarly to intrajejunal levodopa, thus broadening the therapeutic landscape for PD. By bypassing presynaptic terminals and providing steady stimulation of postsynaptic receptors, apomorphine infusion may mimic physiological dopamine tone in motor circuits. This appears to counteract the cortical hyperexcitability and loss of plasticity caused by long-term dopaminergic pulsatility.^50^ Notably, we found that improvements in cortical excitability (e.g., reduced 120% rMT MEP amplitude) were significantly correlated with improvements in clinical motor scores. This suggests that as motor cortex function is normalized, patients experienced better motor performance. The infusion's effects on cortical circuitry can be interpreted in light of dopamine's complex modulation of plasticity. Dopamine acting on D1 receptors is known to facilitate LTP-like excitatory plasticity, whereas D2 receptor activity can promote LTD-like inhibitory plasticity, and an optimal dopaminergic level is required for normal plasticity.^51^ In dopamine-depleted PD brains, continuous apomorphine likely elevates dopaminergic stimulation into a more optimal range throughout the day. This may restore the ability of M1 circuits to undergo plastic changes, as reflected in the recovered iTBS response at 24 weeks.

Previous pharmacological studies in healthy individuals have shown that acute administration of apomorphine can deteriorate LTP and LTD-like plasticity induced by transcranial direct current stimulation or paired associative stimulation.^52^ In contrast, our findings demonstrate that long-term continuous apomorphine infusion restores LTP-like plasticity in PD. This discrepancy may stem from differences in dopaminergic tone and receptor sensitivity: in healthy brains, acute supraphysiological dopamine stimulation may disrupt homeostatic mechanisms, whereas in the dopamine-depleted PD brain, continuous stimulation may re-establish balanced dopaminergic signaling and promote synaptic plasticity through sustained D1/D2 receptor activation.

Finally, our finding that SICI and ICF measures did not differ significantly between PD patients and HC at baseline, and that - as expected - these parameters remained unchanged following apomorphine infusion, appears to contrast with previous studies reporting altered intracortical inhibition and facilitation in PD, which were at least partially responsive to acute levodopa administration.^3,4,10,53,54^ It also differs from observations on intrajejunal levodopa, which has been shown to restore not only cortical excitability and plasticity, but also inhibitory circuits.^20^ However, SICI and ICF have shown greater variability across studies - even when using standardized protocols - compared to measures of excitability and plasticity. This inconsistency may stem from clinical heterogeneity of PD cohorts studied.^2^ Nonetheless, the progressive trend toward normalized excitability observed after apomorphine initiation in our cohort may indicate an indirect rebalancing of intracortical inhibitory-excitatory dynamics on basal ganglia output and thalamo-cortical drive.

### Limitations

Several limitations of this study should be acknowledged. First, the sample size completing follow-up was relatively small (15 PD patients), and there was no parallel control group of PD patients on sham or conventional therapy, which limits generalizability. Future studies with larger cohorts should employ mixed-effects modeling to more fully exploit the longitudinal structure of the data. Furthermore, while our follow-up at 24 weeks indicates sustained benefits, longer-term observations would determine if cortical plasticity enhancements persist or plateau with continued therapy beyond 6 months. It will also be important in future studies to compare continuous apomorphine infusion with other advanced therapies (foslevodopa/foscarbidopa continuous subcutaneous infusion) in head-to-head studies to verify whether they differ in restoring cortical physiology.

## Conclusions

In summary, this study provides novel evidence that continuous dopaminergic stimulation via apomorphine was associated with progressive improvement of selected cortical markers in PD, along with concurrent clinical benefits. These neurophysiological improvements likely underpin the clinical effects and highlight the therapeutic value of addressing the cortical component of PD pathophysiology.

## Supplemental Material

sj-docx-1-pkn-10.1177_1877718X261479077 - Supplemental material for Continuous apomorphine infusion modulates cortical hyperexcitability and abnormal plasticity in Parkinson's disease Supplemental material, sj-docx-1-pkn-10.1177_1877718X261479077 for Continuous apomorphine infusion modulates cortical hyperexcitability and abnormal plasticity in Parkinson's disease by Maria Ilenia De Bartolo, Francesco Marchet, Sara Pietracupa, Carolina Cutrona, Flavia Aiello, Matteo Costanzo, Giorgio Leodori, Giorgio Vivacqua, Antonella Conte, Giovanni Fabbrini, Alfredo Berardelli, Nicola Modugno and Daniele Belvisi in Journal of Parkinson's Disease
