# Supplementary material for: Continuous apomorphine infusion modulates cortical hyperexcitability and abnormal plasticity in Parkinson's disease
Source: J Parkinsons Dis. 2026 Sep 18:1877718X261479077. Online ahead of print. doi: 10.1177/1877718X261479077 (PMC13588967; doi:10.1177/1877718X261479077)
Supplement: Supplementary file 1 [file sj-docx-1-pkn-10.1177_1877718X261479077.docx]

**Supplementary Materials**

**Figure S1. Longitudinal changes in intracortical inhibition and facilitation.** Box plots represent group distributions across four time points (T0, T1, T2, T3) during continuous apomorphine infusion. (A) SICI= Short-interval intracortical inhibition; (B) ICF= Intracortical facilitation. Boxes represent median and interquartile range; whiskers show minimum and maximum values.
